# Transcriptome Analysis Reveals that Vitamin A Metabolism in the Liver Affects Feed Efficiency in Pigs

**DOI:** 10.1534/g3.116.032839

**Published:** 2016-09-14

**Authors:** Yunxia Zhao, Ye Hou, Fei Liu, An Liu, Lu Jing, Changzhi Zhao, Yu Luan, Yuanxin Miao, Shuhong Zhao, Xinyun Li

**Affiliations:** Key Laboratory of Agricultural Animal Genetics, Breeding, and Reproduction of the Ministry of Education, Huazhong Agricultural University, Wuhan 430070, P. R. China; Key Laboratory of Swine Genetics and Breeding of the Ministry of Agriculture, Huazhong Agricultural University, Wuhan 430070, P. R. China; The Cooperative Innovation Center for Sustainable Pig Production, Wuhan 430070, P. R. China

**Keywords:** Feed efficiency, liver, vitamin A metabolism, pig

## Abstract

Feed efficiency (FE) is essential for pig production. In this study, 300 significantly differentially expressed (DE) transcripts, including 232 annotated genes, 28 *cis*-natural antisense transcripts (*cis*-NATs), and 40 long noncoding RNAs (lncRNAs), were identified between the liver of Yorkshire pigs with extremely high and low FE. Among these transcripts, 25 DE lncRNAs were significantly correlated with 125 DE annotated genes at a transcriptional level. These DE genes were enriched primarily in vitamin A (VA), fatty acid, and steroid hormone metabolism. VA metabolism is regulated by energy status, and active derivatives of VA metabolism can regulate fatty acid and steroid hormones metabolism. The key genes of VA metabolism (*CYP1A1*, *ALDH1A2*, and *RDH16*), fatty acid biosynthesis (*FASN*, *SCD*, *CYP2J2*, and *ANKRD23*), and steroid hormone metabolism (*CYP1A1*, *HSD17B2*, and *UGT2B4*) were significantly upregulated in the liver of high-FE pigs. Previous study with the same samples indicated that the mitochondrial function and energy expenditure were reduced in the muscle tissue of high-FE pigs. In conclusion, VA metabolism in liver tissues plays important roles in the regulation of FE in pigs by affecting energy metabolism, which may mediate fatty acid biosynthesis and steroid hormone metabolism. Furthermore, our results identified novel transcripts, such as *cis*-NATs and lncRNAs, which are also involved in the regulation of FE in pigs.

Feed efficiency (FE) is an important economic trait in the pig industry. Feed cost accounts for > 60% of the total production costs. As such, FE should be improved to enhance the benefits of pig production. Thus far, two highly correlated indicators, *i.e.*, feed conversion ratio (FCR) and residual feed intake (RFI), are used to measure FE, and a low FCR/RFI level denotes an improvement in FE (Do *et al.* 2013). Microarray analysis has revealed that lipogenesis and steroidogenesis in liver tissues are related to the FE of pigs (Lkhagvadorj *et al.* 2010). Selection on RFI in growing pigs indicates that catabolic pathway activities in liver and muscle increase in low-FE pigs (Le Naou *et al.* 2012). Meanwhile, the activities of lactate dehydrogenase and β-hydroxylacylCoA dehydrogenase in the liver increase in high-FE pigs (Le Naou *et al.* 2012). In our previous study, the expression of mitochondrial genes was downregulated in the muscle tissue of high-FE pigs (Jing *et al.* 2015). These studies have suggested that the energy metabolism of liver and muscle tissues are essential for the regulation of FE of pigs.

The liver maintains the homeostasis of metabolic processes, including energy and vitamin metabolism. Approximately 70–95% of vitamin A (VA) is stored in the liver of some mammals. VA and its metabolite retinoic acid (RA), which includes all-*trans*-RA (atRA), 9*-cis*-RA, and 13*-cis*-RA etc. (Heyman *et al.* 1992; Lampen *et al.* 2000), modulate energy balance by regulating the function of adipose cells and carbohydrate metabolism (Bonet *et al.* 2012; Obrochta *et al.* 2015). In mice, RA treatment can reduce body fat and improve insulin sensitivity (Berry and Noy 2009; Manolescu *et al.* 2010). In young rats, the synergistic effect of VA and high-fat diet increases adiposity (Redonnet *et al.* 2008). In young ferrets, chronic supplementation with β-carotene, which is a natural material for VA synthesis, increase their body weight and subcutaneous fat mass (Murano *et al.* 2005). The short-term atRA treatment in adulthood is associated with a decrease in adiposity in ferrets (Sánchez *et al.* 2009). The effect of VA and its metabolites on energy metabolism may be dependent on developmental stage.

Molecular mechanism studies have indicated that VA, especially its metabolites, can regulate several genes related to energy metabolism. atRA and 9-*cis* RA can bind to retinoic acid receptors (RARs) with high affinity *in vitro*. The 9-*cis* RA specifically binds to retinoid X receptors (RXRs) *in vitro* (Blomhoff and Blomhoff 2006). RAR:RXR heterodimers regulate typical RA target genes by binding to RA response elements, which include phosphoenolpyruvate carboxykinase (*PEPCK*) (Cadoudal *et al.* 2008) and stearoyl-CoA desaturase 1 (*SCD1*) (Samuel *et al.* 2001; Zolfaghari and Ross 2003). Moreover, RXR can form heterodimers with liver X receptor (LXR), which can positively regulate key lipogenic genes, including *SREBP-1C*, *SCD*, and *FASN*, in mouse liver cells and human hepatoma cells (Mukherjee *et al.* 1997; Roder *et al.* 2007). Therefore, VA and its metabolites play important roles in energy metabolism.

Long noncoding RNAs (lncRNAs) and natural antisense transcripts (NATs) have emerged as essential components of regulatory factors in several physiological processes. lncRNAs are involved in adipogenesis, hepatic lipid metabolism, energy balance, and so on (Zhao and Lin 2015). The knockdown of the liver-enriched lncRNA, *lncLSTR*, can reduce plasma triglyceride levels in a hyperlipidemic mouse model (Li *et al.* 2015). sno-lncRNAs from the Prader-Willi syndrome locus modulate energy balance in mice (Yin *et al.* 2012; Powell *et al.* 2013). In humans, the lncRNA *HULC* modulates lipid metabolism in hepatocellular carcinoma by activating the acyl-CoA synthetase subunit *ACSL1* (Cui *et al.* 2015). In previous studies, NATs, which include *trans*- and *cis*- NATs, have been identified in humans, mice, and pigs, etc. (Katayama *et al.* 2005; Zhang *et al.* 2006; Zhao *et al.* 2016). In eukaryotic, NATs-mediated gene expression regulation mechanisms include chromatin remodeling, transcriptional interference, RNA masking, and double-stranded RNA-dependent mechanisms (Lapidot and Pilpel 2006; Faghihi and Wahlestedt 2009). The antisense transcript of apolipoprotein A1 (*APOA1*), referred to as *ApoA1-AS*, is an lncRNA that negatively regulates *APOA1* expression (Halley *et al.* 2014). However, the roles of lncRNAs and NATs in the FE of pigs remain largely unknown.

On the basis of high-throughput RNA sequencing, we systematically analyzed differentially expressed (DE) annotated genes, lncRNAs, and *cis*-NATs in liver tissues between low- and high-FCR pigs. Gene ontology (GO) and pathway analysis revealed that the VA metabolism pathway in liver tissues was related to the variation of FE in pigs.

## Materials and Methods

### Animals, tissues, and RNA extraction

In this study, the feed intake of 236 purebred castrated boars from a Yorkshire pig population was detected using an ACEMA 64 automated individual feeding system at the Agricultural Ministry Breeding Swine Quality Supervision Inspecting and Testing Center (Wuhan, China) (Jing *et al.* 2015). The FCR of each individual was analyzed in the R environment using the following formula:

$$\mathrm{FCR}=\frac{\text{Feed}\;\text{intake}\;}{\text{Average}\;\text{daily}\;\text{gain}}.$$

Furthermore, the FCR value between the three lowest pigs and the three highest pigs was significantly different (*P* < 0.05) (Jing *et al.* 2015). Those six castrated individuals were selected for RNA sequencing; the selected animals were not sibships. For tissue samples, pigs were slaughtered at 90 kg according to a standard procedure approved by guidelines from Regulation of the Standing Committee of Hubei People’s Congress (Hubei Province, P. R. China). Liver tissues were sampled and snap-frozen in liquid nitrogen within half an hour after slaughter before storage at –80°. Briefly, total RNA was isolated from frozen liver tissues with the TRIzol reagent (Invitrogen). All experimental protocols were approved by the Ethics Committee of Huazhong Agricultural University (HZAUMU2013-0005).

### Library construction and sequencing

For each liver sample, equal quantities of RNA were sent to Genergy Biotechnology (Shanghai, China) for library construction. The RNA-seq library of each sample was prepared by with the TruSeq Stranded Total RNA Sample Preparation kit (Illumina). The Second Strand Marking Mix was also used during second strand cDNA synthesis to replace dTTP with dUTP to ensure strand specificity. After quality control, sequencing was performed with Illumina HiSeq2000.

### RNA sequencing analysis

TopHat (version 2.0.9) (Trapnell *et al.* 2009) software was used to align reads to the pig reference genome (NCBI Sscrofa10.2). Up to two mismatches were allowed in reads mapping. Multiple-mapped reads were discarded; uniquely mapped reads were compared with the gff3 file of the Sscrofa10.2 genome with in-house Perl scripts. The reference genome sequence and its gff3 file were downloaded from the NCBI genome database (ftp://ftp.ncbi.nlm.nih.gov/genomes/Sus_scrofa/). Furthermore, mapped reads in the intergenic region were also compared to annotated lncRNAs (Zhou *et al.* 2014).

### Genome-wide identification of cis-NATs

Mapped reads were pooled into one SAM file, which was used to identify novel transcripts by Cufflinks (Trapnell *et al.* 2012) with the option -library-type fr-firststrand. The transcripts identified by Cufflinks were compared with the gff3 file of the Sscrofa10.2 genome. *Cis*-NATs were identified on the basis of the following criteria: (i) located on the antisense strand of annotated genes, and (ii) novel transcripts but not belonging to the annotated genes. *Cis*-NATs were extracted from novel transcripts by in-house Perl scripts. The identified *cis*-NATs were used for further analysis.

### Differential expression analysis

The count of reads located in the exon regions of each annotated gene, *cis*-NAT, and lncRNA was calculated using in-house Perl scripts. Genes with CPM (at least one count per million) > 1 in at least four samples were kept for further analysis. Both normalization of expression profiling for all expressed transcripts, and identification of DE transcripts were performed by edgeR (Robinson *et al.* 2010) in the *R* environment. Transcripts were determined as significantly DE with FDR < 0.05, and with an upregulated or downregulated fold-change (FC) of ≥ 2.5 between low-FCR and high-FCR pigs.

### Correlation analysis

Pearson correlation analysis was performed to identify the correlatively expressed SA (sense–antisense) pairs and DE annotated gene–lncRNA pairs in the R environment. The *cis*-NATs, annotated genes, and lncRNAs that were detected at least in four samples were chosen for correlation analysis. The criteria for significantly correlated SA pairs was *P* < 0.05 and |R| > 0.8, whereas those for lncRNA-annotated gene pairs was *P* < 0.01 and |R| > 0.95.

### q-PCR validation of DE genes

Total RNA was reverse-transcribed into cDNA using a PrimeScript RT reagent kit (Takara Bio Inc.). Oligonucleotide primers for six DE genes were designed with oligo7 software; the primer sequences are available in Supplemental Material, Table S1. Relative expression levels of these genes in liver were quantified by qPCR. The reactions were conducted on a BIO-RAD CFX384 Real-Time System with SYBR Green PCR Master Mix (Bio-Rad) as described in the manufacturer’s instruction manual. The 10 μl reaction mixture consisted of 1 μl cDNA, with 5 μl 2× SYBR Green PCR Master Mixture, 0.2 μl each of the forward and reverse primers, and 3.6 μl of RNase-free water. Samples were preincubated at 95° for 3 min, followed by 40 PCR amplification cycles (denaturation: 95° for 20 sec, annealing: 60° for 20 sec, elongation: 72° for 15 sec). A dissociation curve was generated at the end of the last cycle by collecting the fluorescence data from 60° to 95°. Relative gene expression levels were normalized to the *RPL32* gene, which has stable expression in liver tissues (Ostrowska *et al.* 2014), by the 2^−ΔΔCt^ method. Student’s *t*-test was used to analyze the expression difference between the low-FCR and high-FCR groups.

### Gene ontology enrichment and pathway analysis

GO enrichment analysis was performed with DAVID Bioinformatics Resources 6.7 (Da Wei Huang and Lempicki 2008). DE genes were sorted by their involvement in significantly enriched biological process GO terms.

The pathway that involved the significant GO-terms-enriched genes was structured by referring to the KEGG pathway database and published articles. *cis*-NATs and DE lncRNAs that were correlated with the expression of genes involved in structured pathways, were also investigated and displayed. Visualization of pathway analysis was implemented in Cytoscape (Saito *et al.* 2012). The log_2_FC of annotated genes, *cis*-NATs, and lncRNAs was calculated as

$$\log_{2}\mathrm{FC}=\log_{2}\left(\text{Low}/\text{High}\right),$$

where Low and High represented expression profile of the low-FCR and high-FCR groups, respectively.

### Data availability

The raw data of RNA-seq were submitted to NCBI Sequence Read Archive database (SRA) under series SRP076030 which will be release in June 2017. DE transcripts list can be found in File S2.

## Results

### RNA sequencing data mapping and annotation

**Figure 1:**
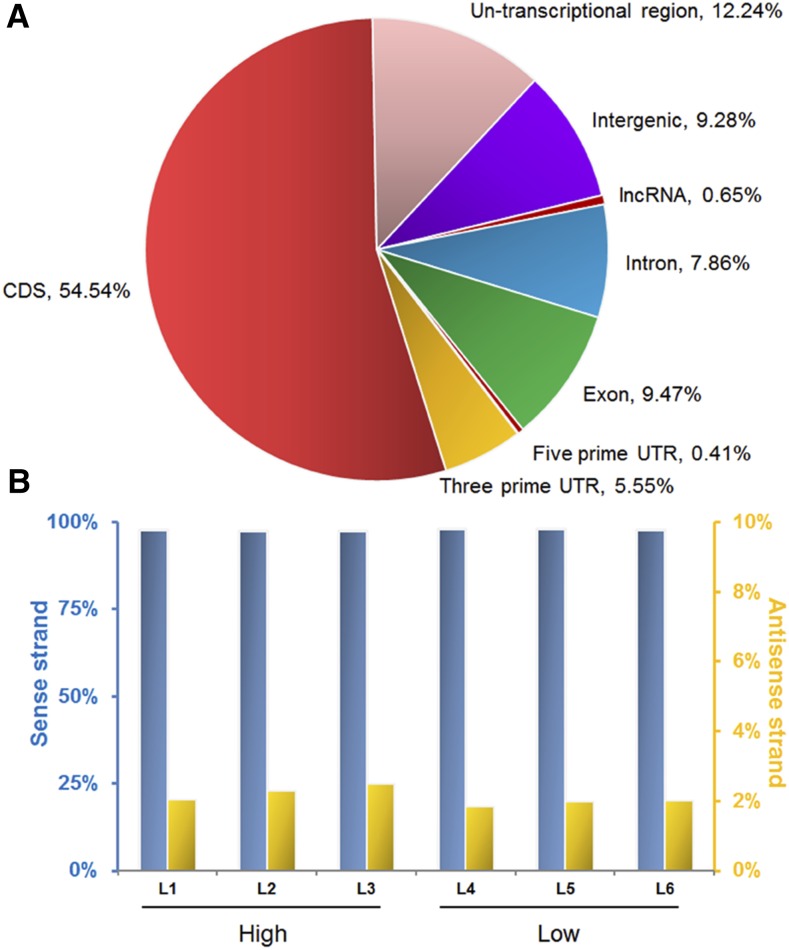
Annotation of the uniquely mapped reads of RNA-seq in liver tissue of pigs. (A) Distribution of uniquely mapped reads in the pig genome. The percentages in this pie chart represent the mean of all six RNA-seq data. On average, over 50% uniquely mapped reads were located in the CDS region. The untranscriptional regions were defined as genome region of annotated genes but without coverage of annotated transcripts. (B) Reads distributed on the sense and antisense strands of annotated genes. Approximately 2% reads were mapped on antisense strands of annotated genes.

RNA of liver tissues of three high-FCR (H) and three low-FCR (L) pigs was isolated for RNA-seq. The RNA-seq raw data have been submitted to the NCBI Sequence Read Archive (SRA) under series SRP076030. After removing adaptors and filtering low quality reads, 7.1–10.0 million clean reads were yielded (Table 1). In these clean reads, ∼87% (6.1–9.6 million) were mapped on the Sscrofa10.2 genome, and over 90% of them were unique mappings (Table 1). The majority of uniquely mapped reads (90.72%) were located in the annotated gene region, whereas > 50% were located in the CDS region (Figure 1A). However, nearly 20% uniquely mapped reads were located in the intron region (9.28%), or other nontranscriptional regions (12.24%; Figure 1A). We also identified 1.8–2.5% uniquely mapped reads that were distributed on the antisense strand of annotated genes (Figure 1B).

**Table 1:** Summary of RNA-seq data from six liver samples

Group	Sample	Input	Mapped	Uniquely mapped	Multiple mapped
High	L1	11,028,888	9,662,607 (87.61%)	9,023,627 (93.39%)	638,980 (6.61%)
	L2	8,901,564	7,827,119 (87.93%)	7,076,183 (90.41%)	750,936 (9.59%)
	L3	8,551,718	7,670,784 (89.70%)	7,038,474 (91.76%)	632,310 (8.24%)
Low	L4	7,148,669	6,283,279 (87.89%)	5,677,878 (90.36%)	605,401 (9.64%)
	L5	8,324,699	7,348,705 (88.28%)	6,797,361 (92.50%)	551,344 (7.50%)
	L6	7,155,132	6,191,448 (86.53%)	5,741,224 (92.73%)	450,224 (7.27%)

### Identification of cis-NATs

Based on the strand-specific RNA-seq reads, *cis*-NATs could be identified directly. Reads that were mapped on the antisense strand of the annotated gene were identified and defined as antisense reads. A total of 0.78 million antisense reads were identified from the uniquely mapped reads. Finally, 1769 *cis*-NATs were identified (File S1).

**Figure 2:**
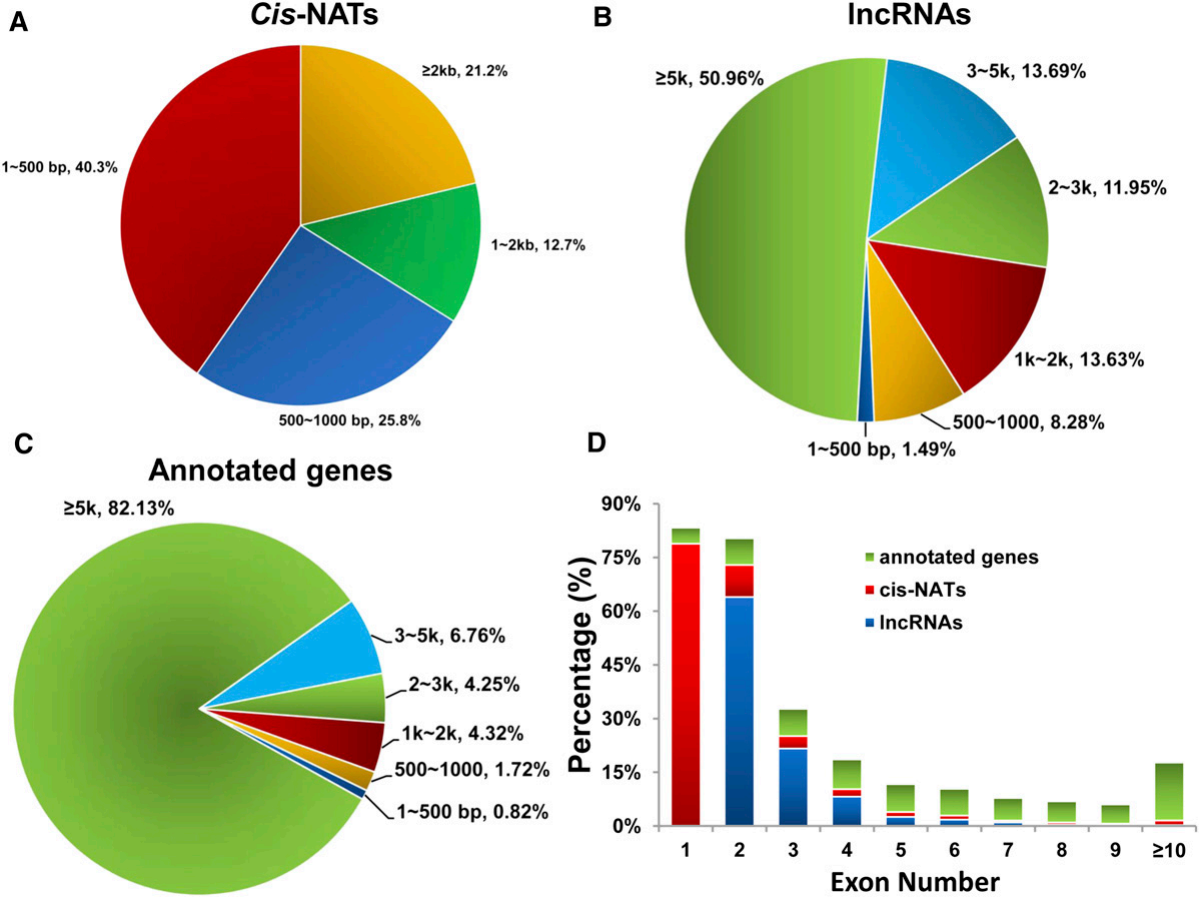
Characteristics of *cis*-NATs, lncRNAs, and annotated genes expressed in collected liver tissues of six pigs. (A) Length distribution of identified *cis*-NATs. The most enriched length of *cis*-NATs was < 500 bp. (B) Length distribution of expressed lncRNAs. Almost 50% expressed lncRNAs were < 5 kb. (C) Length distribution of expressed annotated genes. (D) Statistical results of exon number for expressed *cis*-NATs (red), lncRNAs (blue), and annotated genes (green).

To understand the structural characteristics of the *cis*-NATs, we further investigated their length distribution and exon number. Statistical results showed that the majority (78.80%) of *cis*-NATs were shorter than 2 kb (Figure 2A). The statistical results of reported lncRNAs showed that ∼50% of the lncRNAs were shorter than 5 kb (Figure 2B). Both *cis*-NATs and lncRNAs differed with the detected annotated genes. For the annotated genes, > 80% were > 5 kb (Figure 2C). Moreover, the overwhelming majority of *cis*-NATs (91.22%) and lncRNAs (93.84%) contained fewer than three exons (Figure 2D). Besides, 78.83% of *cis*-NATs contained only one exon (Figure 2D). Most of the detected annotated genes contained more than three exons (Figure 2D). Therefore, the structural characteristics of *cis*-NATs were similar to those of lncRNAs, and both of them were different from the detected annotated genes.

### DE liver transcripts between high and low FCR pigs

**Figure 3:**
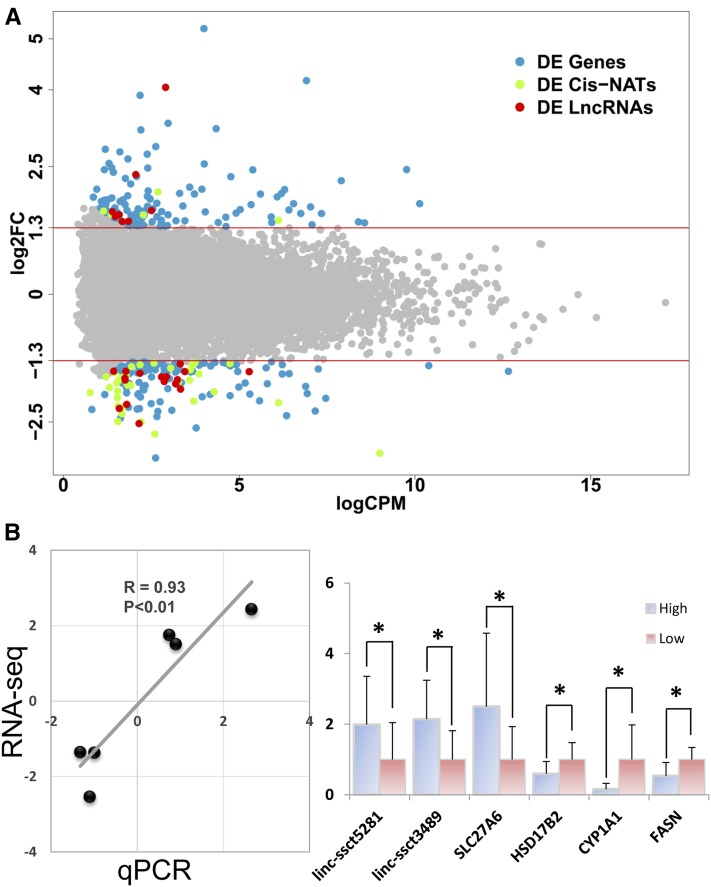
DE transcripts between low- and high-FCR pigs. (A) Plot of the DE transcripts with FC ≥ 2.5 and FDR ≤ 0.05. Light blue, light green, and red dots represented the DE annotated genes, *cis*-NATs and lncRNAs, respectively. The *x*-axis and *y*-axis represent the logCPM and log_2_FC, respectively. (B) qPCR validation of DE transcripts in liver tissue of high-FCR (*n* = 10) and low-FCR (*n* = 10) pigs. The left plot is correlation analysis of RNA-seq and qPCR. For right bar plot, qPCR data were represented as mean ± SD; * *P* < 0.05.

To compare the transcriptome alteration between high- and low-FCR pigs, the DE annotated genes, *cis*-NATs, and lncRNAs were determined using the edgeR package. A total of 300 significantly DE transcripts were identified, which included 232 annotated genes, 28 *cis*-NATs, and 40 lncRNAs, respectively (up or downregulated FC ≥ 2.5 and FDR < 0.05; File S2). Among these 232 annotated genes, 126 genes were upregulated, and 106 genes were downregulated in the low-FCR pigs (Figure 3 and File S2). Moreover, the majority of the DE *cis*-NATs (35 of 40) and lncRNAs (20 of 28) were downregulated in the low-FCR pigs (Figure 3 and File S2). The top 10 DE annotated genes *cis*-NATs and lncRNAs are listed in Table 2.

**Table 2:** DE transcripts between low- and high-FCR groups (top 10)

Source	ID	Gene Name	Log2FC (Low/High)	*P*-Value	FDR
Annotated genes	NM_001167795.1	*ACTA1*	5.20	7.39E-31	4.98E-27
	NM_001243319.1	*IGLC*	4.18	1.40E-33	1.89E-29
	NM_213855.1	*MYH7*	3.89	5.04E-14	8.48E-11
	XM_001924431.4	*ASAH2*	3.35	4.71E-15	9.05E-12
	XM_003126676.2	*MYBPC1*	3.22	2.02E-11	1.43E-08
	XM_001924841.4	*ELOVL2*	−2.38	8.84E-14	1.32E-10
	XM_003359949.1	*TP53INP2*	−2.39	6.44E-09	2.22E-06
	XM_003359189.2	*ERO1LB*	−2.43	1.46E-06	2.46E-04
	XM_003133832.3	*KIF1A*	−2.62	7.90E-13	7.59E-10
	XM_001929103.3	*PARP6*	−3.20	6.13E-13	6.87E-10
*Cis*-NATs	XM_001925338.4-AS1.1	*A2ML1*	2.00	4.58E-07	8.98E-05
	XM_003127358.3-AS1.1	*LOC100524748*	1.63	2.12E-03	4.75E-02
	XM_003126577.2-AS1.1	*CCDC77*	1.57	1.64E-03	4.03E-02
	XM_003359961.2-AS1.1	*LOC100622161*	1.55	1.48E-04	8.60E-03
	NM_214056.1-AS3.1	*MT-III*	1.45	2.65E-06	3.81E-04
	XM_003358629.1-AS2.3	*CCNL1*	−2.34	2.27E-06	3.40E-04
	XM_001926397.3-AS7.1	*FAM135B*	−2.49	1.40E-06	2.41E-04
	XM_003480614.1-AS1.1	*LOC100736683*	−2.50	2.51E-08	6.75E-06
	XM_003122843.3-AS1.1	*DGKZ*	−2.74	1.54E-10	8.64E-08
	XM_003481282.1-AS1.1	*LOC100739207*	−3.11	3.34E-22	1.50E-18
lncRNA	linc-ssct5500	*linc-ssct5500*	4.05	1.75E-18	4.72E-15
	linc-ssct3976	*linc-ssct3976*	2.34	2.31E-07	5.02E-05
	linc-ssct3660	*linc-ssct3660*	1.64	3.67E-05	3.12E-03
	linc-ssct5436	*linc-ssct5436*	1.62	1.10E-03	3.18E-02
	linc-ssct5501	*linc-ssct5501*	1.56	9.53E-04	2.99E-02
	linc-ssct6319	*linc-ssct6319*	−1.76	1.56E-06	2.54E-04
	linc-ssct6159	*linc-ssct6159*	−1.86	3.16E-07	6.54E-05
	linc-ssct1651	*linc-ssct1651*	−2.16	5.59E-06	6.97E-04
	linc-ssct6167	*linc-ssct6167*	−2.24	7.63E-06	8.91E-04
	linc-ssct3489	*linc-ssct3489*	−2.53	2.40E-08	6.67E-06

qPCR was performed to validate the DE transcripts that were identified by RNA-seq. A total of 10 low-FCR individuals and 10 high-FCR individuals, which contained the individuals for RNA-seq, were chosen for qPCR analysis. The difference between these low- and high-FCR pigs was significant. Here, six DE transcripts were chosen for qPCR analysis. Three DE genes (*HSD17B2*, *CYP1A1*, and *FASN*) were selected from the GO enrichment analysis, and another three DE transcripts (*SLC27A6*, *linc-ssct5281*, and *linc-ssct3489*) were selected randomly from DE analysis results (File S2). The qPCR results shows that all six selected genes were validated as significantly DE genes in low-FCR *vs.* high-FCR. Moreover, the correlation coefficient of the log_2_FC values between RNA-seq and qPCR was 0.93 (*P* < 0.05).

### Identification of correlated expression pairs

**Figure 4:**
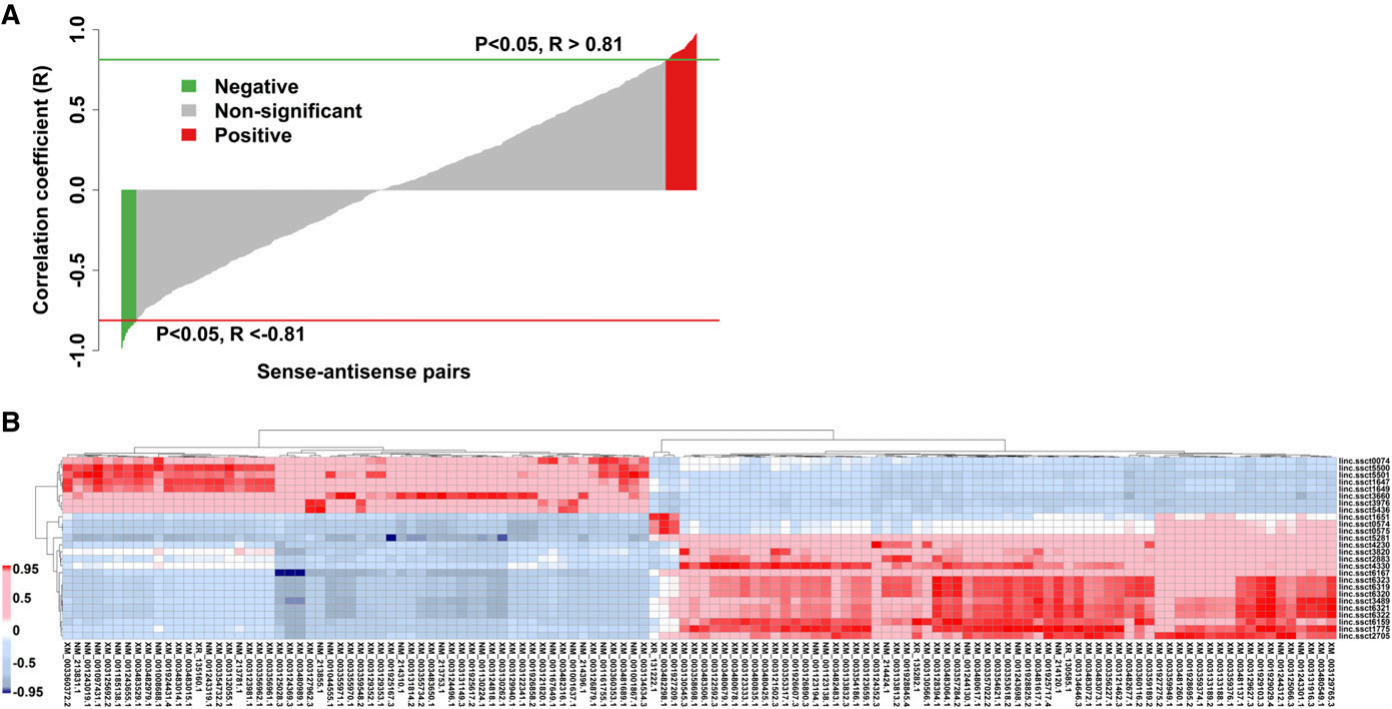
Representation of correlation analysis. (A) Correlation analysis between *cis*-NATs and their sense genes. The red and green bar plots represent the positively and negatively correlated *cis*-NATs and sense genes pairs, respectively. (B) Heat map of correlation analysis between DE lncRNAs and annotated genes. The red and blue fill colors of the heat map cells represent the positive and negative correlations, respectively. The color bar is shown on the left.

To understand function of *cis*-NATs, correlation analysis was performed between the expression of *cis*-NATs and their corresponding sense annotated genes. A total of 91 *cis*-NATs were significantly (*P* < 0.05 and |R| > 0.8) correlated with the expression of their sense annotated genes (Figure 4A and File S3). Among these pairs, 61 (67.4%) were positively correlated (Figure 4A and File S3).

We also analyzed the correlation between the expression of DE lncRNAs and their annotated genes. In total, 337 significant correlation pairs were identified (*P* < 0.01 and |R| > 0.95), which included 26 DE lncRNAs and 126 DE annotated genes (Figure 4B and File S3). Furthermore, the majority (19 in 25) of the lncRNAs were correlated with at least two annotated genes (Figure 4B and File S3). Moreover, 333 of 337 correlated pairs (98.81%) were positively correlated.

### Function annotation

**Figure 5:**
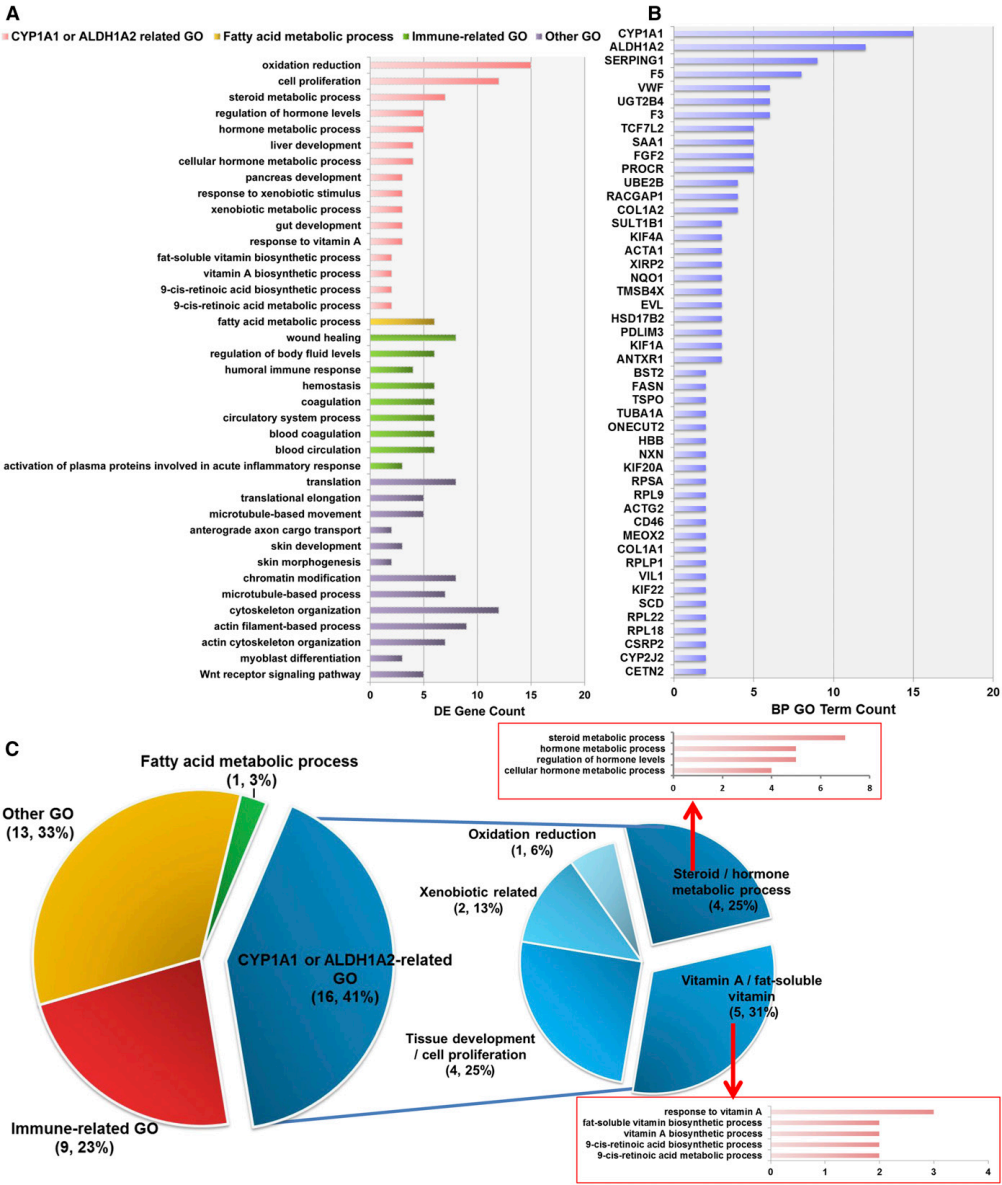
Significantly enriched GO terms for biological processes. (A) Significantly enriched biological process GO terms. The significant GO terms were determined by DAVID Bioinformatics Resources 6.7 (EASE Score < 0.1). Pink, orange, green, and purple colors represented the *CYP1A1*- or *ALDH1A2*-related, fatty acid metabolic process, immune-related, and other GO terms, respectively. (B) Distribution of DE genes in significantly enriched GO terms. Genes involved in at least two GO terms are listed. (C) Statistical analysis of the clustering results of GO terms. The most enriched category was the *CYP1A1*- or *ALDH1A2*-related GO terms (41%; left pie plot), which included five vitamin processes and four steroid-/hormone-related processes (right pie plot).

Function enrichment analysis was performed to detect significant biological processes GO terms of the DE annotated genes and *cis*-NATs according to the DAVID Bioinformatics Resources 6.7. A total of 39 significantly (EASE Score < 0.1) enriched GO terms were identified (Figure 5A and Table S2). The GO enrichment results showed the GO terms could be clustered based on the same DE annotated genes or similar biological functions (Figure 5, A and B). Based on that, the resultant 39 GO terms were clustered into four categories: *CYP1A1*- or *ALDH1A2*-related, fatty acid metabolic processes, immune-related, and other GO terms (Figure 5, A and C, left pie plot). Furthermore, 11 of 16 *CYP1A1*- or *ALDH1A2*-related GO terms contained both genes. Distribution investigation of annotated genes, among significantly GO terms, showed that *CYP1A1* and *ALDH1A2* were the top two most enriched genes (Figure 5B). Each was involved at least 12 GO terms (Figure 5B). Moreover, five of the 16 *CYP1A1*- or *ALDH1A2*-related GO terms were related to vitamin metabolism (Figure 5C).

### Pathway analysis of DE genes and lncRNAs

**Figure 6:**
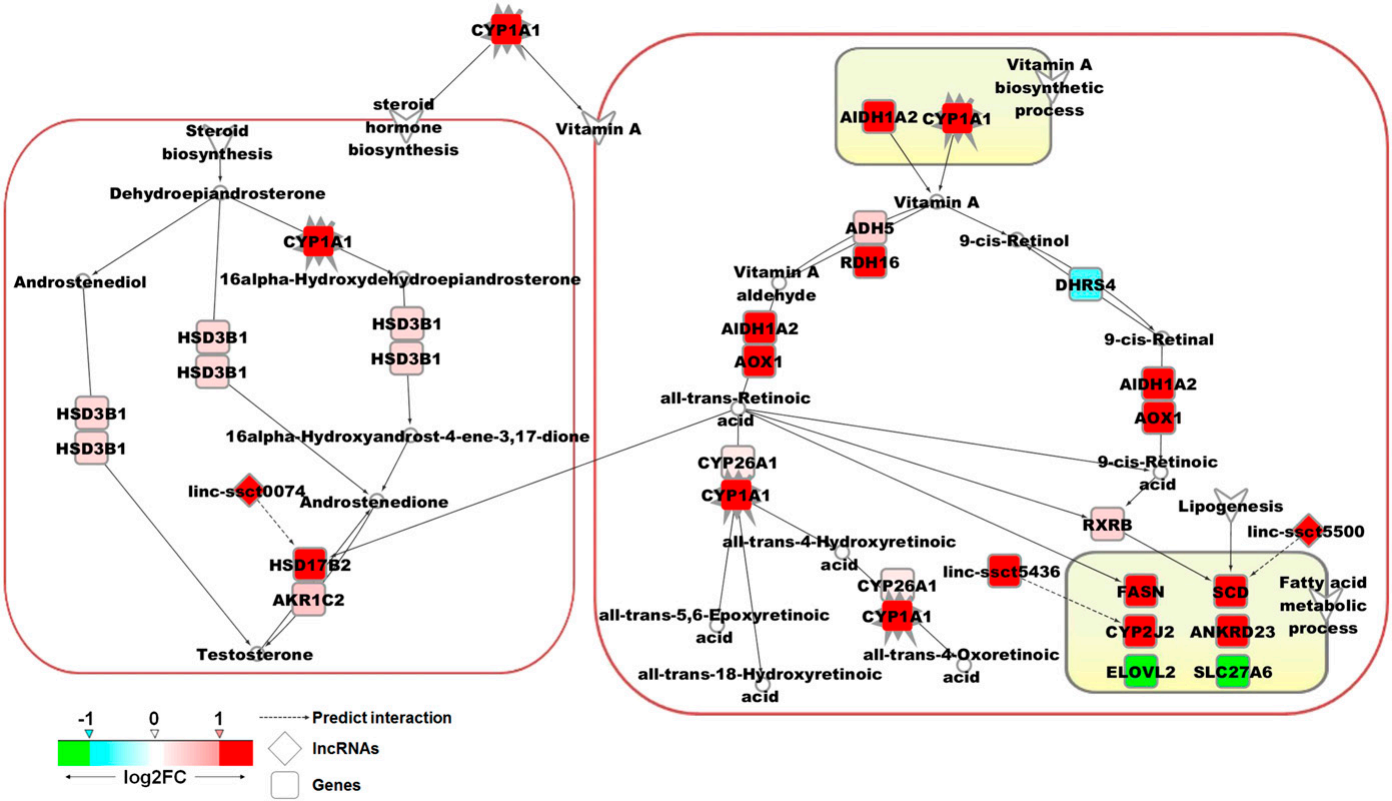
Potential pathway of the annotated DE genes and lncRNAs in the liver tissues of high and low FCR pigs. Red and pink indicates upregulation in low FCR pigs (red, log2FC > 0.5; pink, 0 < log2FC ≤ 0.5); green and blue indicates downregulation in low FCR pigs (green, log_2_FC < −0.5; blue, −0.5 ≤ log_2_FC < 0).

Based on functional enrichment analysis, *CYP1A1* and *ALDH1A2* were the two most important genes, mainly participating in VA- and hormone-related processes. Furthermore, we drew the VA and steroid hormone metabolism pathways according to the KEGG pathway database and previous studies. The results showed that *CYP1A1*, *ALDH1A2*, and *RDH16* were both involved in VA anabolism and catabolism (Figure 6, right). Moreover, fatty acid and steroid hormone metabolic processes were located downstream of the VA metabolism pathway. These processes were regulated by RA, which is a metabolite of VA. The varied expression of the key genes and lncRNAs involved in these pathways were labeled by different colors. In Figure 6, genes in red and pink were upregulated in low-FCR pigs, whereas genes in green and blue were downregulated. The *CYP1A1*, *ALDH1A2*, *RDH16*, *HSD17B2*, *UGT2B4*, *FASN*, *SCD*, *CYP2J2*, and *ANKRD23* genes were upregulated, in low-FCR pigs; the DE lncRNAs *linc-ssct5500*, *linc-ssct5436*, and *linc-ssct0074* were also positively correlated with *SCD*, *CYP2J2*, and *HSD17B2*, respectively (Figure 6). Other VA metabolism-involved genes (ADH5, CYP26A1, *AOX1*, and *RXRB*) were also slightly upregulated in low-FCR pigs.

## Discussion

The improvement of FE is one of the most efficient ways to improve the benefits of pig production. Thus, investigation of the mechanisms of FE is very important for pig breeding. In this study, we systematically analyzed the transcription profiles of liver tissue in high- and low-FCR pigs. We found that the VA metabolism pathway in liver tissues was important for FE in pigs. The upregulation of genes related to VA metabolism in the liver tissue was positively correlated with FE in pigs.

The liver is one of the main tissues for VA storage in mammals. In this study, the key genes of VA metabolism, namely, *CYP1A1*, *ALDH1A2*, and *RDH16*, were all significantly upregulated in the liver of high-FE (low-FCR) pigs. This result indicated that the pathways of VA metabolism differed between high- and low-FE pigs. A recent study indicated that RA biosynthesis is regulated by energy status at the rate-limiting step catalyzed by retinol dehydrogenases (*RDH*) (Obrochta *et al.* 2015). The retinol dehydrogenase family members *RDH1*, *RDH10*, and *RDH16* had significantly decreased transcriptional levels after refeeding, oral gavage with glucose, or injection with insulin (Obrochta *et al.* 2015). These results implied a negative relationship between RA biosynthesis and energy levels. Moreover, previous studies indicated that mitochondrial reactive oxygen species, and mitochondrial uncoupling reactions involving genes of high-FE pigs, were lower than those of low-FE pigs (Grubbs *et al.* 2013a; Jing *et al.* 2015). However, the antioxidant defenses of high-FE pigs were higher than those of low-FE pigs (Grubbs *et al.* 2013b). The oxidation and energy loss of high-FE pigs was lower than that of low-FE pigs. Thus, the upregulation of genes involving in VA metabolism in high-FE pigs was possibly caused by the relatively lower oxidation and energy loss in this study. Besides, the key genes of fatty acid biosynthesis *SCD* (Samuel *et al.* 2001; Zolfaghari and Ross 2003; Zhang *et al.* 2012; Weiss *et al.* 2014) and *FASN* (Zhou *et al.* 2010; Zhang *et al.* 2012) were induced by RA at the transcriptional level and significantly upregulated in high-FE pigs. *SCD* encodes stearoyl-CoA desaturase, which converts saturated fatty acids into monounsaturated fatty acids, and influences fatty acid partitioning in the liver by promoting fatty acid synthesis but decreasing oxidation (Flowers and Ntambi 2009). Furthermore, other genes related to fatty acid biosynthesis, namely, *CYP2J2* and *ANKRD23* were also upregulated. These results indicated that fatty acid biosynthesis in the liver was increased in high-FE pigs. Therefore, the VA pathway could affect FE by involving the energy metabolism of pigs and promoting fatty acid biosynthesis. Moreover, the moderate activity of the VA metabolic pathway in liver tissue may benefit FE in pigs.

Previous studies also indicated that RA could induce and maintain testosterone production (Tucci *et al.* 2008). Testosterone is a steroid hormone in vertebrates and belongs to the androgen group. This hormone plays a key role in male reproductive tissue development, as well as in increasing muscle mass, muscle strength, bone mass, and bone mineral density (van den Beld *et al.* 2000; Sinha-Hikim *et al.* 2002). In this study, genes involved in testosterone metabolism, namely, *CYP1A1*, *HSD17B2*, and *UGT2B4*, were significantly upregulated in low-FCR pigs. Moreover, *HSD17B2* is positively regulated by RA (Ito *et al.* 2001). *HSD3B1* and *AKR1C2* are involved in testosterone metabolism. and were also slightly upregulated in high-FE pigs. These results suggested that the metabolism of testosterone was increased in high-FE pigs; VA may also affect FE by affecting steroid hormone metabolism.

In addition, 40 lncRNAs were differentially expressed between high- and low-FCR pigs. lncRNAs are involved in several bioprocesses, including energy metabolism (Yin *et al.* 2012; Powell *et al.* 2013; Cui *et al.* 2015; Li *et al.* 2015) and growth (Dey *et al.* 2014; Mueller *et al.* 2015). The DE lncRNAs *linc-ssct5500*, *linc-ssct5436*, and *linc-ssct0074* were upregulated in the livers of low-FCR pigs, and positively correlated with *SCD*, *CYP2J2*, and *HSD17B2*, respectively. Previous studies indicated that *SCD* and *CYP2J2* (Wang *et al.* 2004) are involved in fatty acid metabolism, whereas *HSD17B2* is related to testosterone metabolism (Labrie *et al.* 1997). Therefore, these lncRNAs may also affect the FE of pigs by altering the metabolism of fatty acids and steroid hormones.

We also identified 1769 *cis*-NATs in pig liver tissue. Among them, 28 were significantly different between low and high FE pigs. Correlation analysis showed 61 (67.4%) SA pairs from 91 significantly correlated SA pairs were positively correlated. The coexpression (positively) between SA pairs was reported in humans (Chen *et al.* 2005), *Drosophila melanogaster* (Okamura *et al.* 2008), and pigs (Zhao *et al.* 2016). These results indicate that *cis*-NATs are involved in the regulation of FE of pigs. However, the mechanisms of the regulatory roles of *cis*-NATs in FE of pigs remained largely unknown. Furthermore, some immune-related signaling pathways were also identified to relate to FE. Therefore, the FE of pigs may also be regulated by ncRNAs or other pathways.

In summary, annotated DE genes, *cis*-NATs, and lncRNAs in the liver tissues of high- and low-FE pigs were analyzed comparatively. Our results revealed that different expression was enriched mainly in VA, fatty acid, and steroid hormone metabolism. VA metabolism in liver tissues involved regulation of FE in pigs by mediating fatty acid biosynthesis and steroid hormone metabolism. *CYP1A1*, *ALDH1A2*, and *RDH16* are the key upstream genes of VA metabolism, and possible candidate genes for FE in pigs. Some lncRNAs and *cis*-NATs were also related to the FE of pigs.

## Supplementary Material

Supplemental Material
