# Supplementary material for: Transcriptome Analysis Reveals that Vitamin A Metabolism in the Liver Affects Feed Efficiency in Pigs
Source: G3 (Bethesda). 2016 Sep 14;6(11):3615–24. doi: 10.1534/g3.116.032839 (PMC5100860; doi:10.1534/g3.116.032839)
Supplement: Supplemental Material [file supp_6_11_3615__index.html]

Transcriptome Analysis Reveals that Vitamin A Metabolism in the Liver Affects Feed Efficiency in Pigs — Supplemental Material 

# Transcriptome Analysis Reveals that Vitamin A Metabolism in the Liver Affects Feed Efficiency in Pigs

## Supplemental Material for Zhao *et al.*, 2016

**Files in this Data Supplement:**

- Table S1 - PCR primers of 6 selected different expressed transcripts. (.pdf, 75 KB)
- Table S2 - Significantly enriched GO terms of different expressed genes. (.pdf, 226 KB)
- File S1 - Gff file of antisense transcript. (.xls, 766 KB)
- File S2 - DE transcripts list. (.xls, 66 KB)
- File S3 - Significant correlation pairs. (.xls, 80 KB)
