# Supplementary material for: Transcriptome Analysis Reveals that Vitamin A Metabolism in the Liver Affects Feed Efficiency in Pigs
Source: G3 (Bethesda). 2016 Sep 14;6(11):3615–24. doi: 10.1534/g3.116.032839 (PMC5100860; doi:10.1534/g3.116.032839)
Supplement: Supplemental Material [file supp_g3.116.032839_TableS1.pdf]

Table S1 PCR primers of 6 selected different expressed transcripts

Number	Symbol	Primer sequence 5′–3′	Product length (bp)	Tm (℃)
Linc-ssct5281	F	GTGGCTCTGGGATGAATGCT	128 bp	60
	R	GGGGGCGTGGTAAATGAAGA		
Linc-ssct3489	F	GGGGAGCAGCCAGTACAAG	100 bp	60
	R	CCGTCTACGGAGGATAACTGAC		
SLC27A6	F	TGGGTGCAGATTTGCTTGGA	227 bp	60
	R	TGGTTTGGGGAGACCTGTTG		
HSD17B2	F	ATTCTGGGATTGGCCATGCT	166 bp	60
	R	CAAGGATCCCTGCGTTGTTG		
CYP1A1	F	GTGGCTCTGGGATGAATGCT	198 bp	60
	R	GGCATTCTCGTCCATCCTCT		
FANS	F	CCTGCCACAACTCCAAAGACA	139 bp	60
	R	ATGAAGTAGGAGTGGAAGGCGA		

F, forward; R, reverse.
