# Supplementary material for: Transcriptome Analysis Reveals that Vitamin A Metabolism in the Liver Affects Feed Efficiency in Pigs
Source: G3 (Bethesda). 2016 Sep 14;6(11):3615–24. doi: 10.1534/g3.116.032839 (PMC5100860; doi:10.1534/g3.116.032839)
Supplement: Supplemental Material [file supp_g3.116.032839_TableS2.pdf]

Table S2 Significantly enriched GO terms of different expressed genes

ID	Term	Count	%	PValue	Genes
GO:0016055	Wnt receptor signaling pathway	5	0.20	0.07	TCF7L2, UBE2B, NXN, NLK, PYGO1
GO:0045445	myoblast differentiation	3	0.12	0.02	TCF7L2, CSRP2, LGALS1
GO:0030036	actin cytoskeleton organization	7	0.28	0.05	RACGAP1, ANTXR1, PDLIM3, EVL, TMSB4X, XIRP2, ACTA1
GO:0030029	actin filament-based process	9	0.36	0.01	RACGAP1, ANTXR1, PDLIM3, EVL, TMSB4X, XIRP2, ACTA1, VIL1, MYH7
GO:0007010	cytoskeleton organization	12	0.48	0.01	RACGAP1, UBE2B, ANTXR1, PDLIM3, EVL, TMSB4X, XIRP2, ACTA1, CETN2, VIL1, KRT8, ANK3
GO:0007017	microtubule-based process	7	0.28	0.07	UBE2B, KIF1A, KIF4A, CETN2, KIF22, KIF20A, TUBA1A

GO:0016568	chromatin modification	8	0.32	0.04	UBE2B, RBBP4, EYA3, ARID1A, DPF3, HDAC11, CHD6, SETD7
GO:0043589	skin morphogenesis	2	0.08	0.05	COL1A2, COL1A1
GO:0043588	skin development	3	0.12	0.04	COL1A2, COL1A1, LTB
GO:0008089	anterograde axon cargo transport	2	0.08	0.07	KIF1A, KIF4A
GO:0007018	microtubule-based movement	5	0.20	0.04	KIF1A, KIF4A, KIF22, KIF20A, TUBA1A
GO:0006414	translational elongation	5	0.20	0.03	RPL18, RPL22, RPLP1, RPL9, RPSA
GO:0006412	translation	8	0.32	0.09	RPL18, RPL22, RPLP1, RPL9, RPSA, MRPL51, RARS2, NACA
GO:0006959	activation of plasma proteins involved in acute inflammatory response	3	0.16	0.06	SERPING1, CD46, BST2, IL7
GO:0002541	blood circulation	6	0.12	0.09	SERPING1, F3, CD46
GO:0003013	blood coagulation	6	0.24	0.06	SERPING1, F5, COL1A2, MEOX2, ACTG2, HBB
GO:0008015	circulatory system process	6	0.24	0.06	SERPING1, F5, COL1A2, MEOX2, ACTG2, HBB
GO:0042060	coagulation	6	0.32	0.01	SERPING1, F5, F3, VWF, PROCR, FGF2, SAA1, ENO3
GO:0007596	hemostasis	6	0.24	0.01	SERPING1, F5, F3, VWF, PROCR, SAA1
GO:0007599	humoral immune response	4	0.24	0.01	SERPING1, F5, F3, VWF, PROCR, SAA1
GO:0050878	regulation of body fluid levels	6	0.24	0.02	SERPING1, F5, F3, VWF, PROCR, SAA1
GO:0050817	wound healing	8	0.24	0.01	SERPING1, F5, F3,

					VWF, PROCR, SAA1
GO:0006631	fatty acid metabolic process	6	0.24	0.08	CYP2J2, SCD, FASN, SLC27A6, ANKRD23, ELOVL2
GO:0042905	9-cis-retinoic acid metabolic process	2	0.08	0.05	CYP1A1, ALDH1A2
GO:0042904	9-cis-retinoic acid biosynthetic process	2	0.08	0.05	CYP1A1, ALDH1A2
GO:0035238	vitamin A biosynthetic process	2	0.08	0.05	CYP1A1, ALDH1A2
GO:0042362	fat-soluble vitamin biosynthetic process	2	0.08	0.06	CYP1A1, ALDH1A2
GO:0033189	response to vitamin A	3	0.12	0.08	CYP1A1, ALDH1A2, HSD17B2
GO:0048565	gut development	3	0.12	0.06	CYP1A1, ALDH1A2, TCF7L2
GO:0006805	xenobiotic metabolic process	3	0.12	0.02	CYP1A1, UGT2B4, NQO1
GO:0009410	response to xenobiotic stimulus	3	0.12	0.03	CYP1A1, UGT2B4, NQO1
GO:0034754	pancreas development	3	0.16	0.03	CYP1A1, ALDH1A2, UGT2B4, FGF2
GO:0001889	cellular hormone metabolic process	4	0.16	0.02	CYP1A1, ALDH1A2, VWF, ONECUT2
GO:0042445	liver development	4	0.20	0.03	CYP1A1, ALDH1A2, UGT2B4, FGF2, SULT1B1
GO:0010817	hormone metabolic process	5	0.20	0.10	CYP1A1, ALDH1A2, UGT2B4, FGF2, SULT1B1
GO:0008202	regulation of hormone levels	5	0.28	0.03	CYP1A1, UGT2B4, FGF2, HSD17B2, SULT1B1, TSPO, NR5A2
GO:0008283	steroid metabolic process	7	0.48	0.01	CYP1A1, TCF7L2, RACGAP1, CSRP2, TSPO, BST2,

GO:0055114	cell proliferation	12	0.60	0.02	LAMP3, PA2G4, CDK9, DLGAP5, LGR4, PRG4 CYP1A1, ALDH1A2, F5, HSD17B2, NQO1, CYP2J2, SCD, NXN, FASN, GLYR1, ERO1LB, KCNAB1, ADI1, RDH16, DHODH
GO:0031016	oxidation reduction	15	0.12	0.06	ALDH1A2, TCF7L2, ONECUT2
